# Convenience behavior in German university students is associated with sociodemographic, study- and health-related factors

**DOI:** 10.3389/fpubh.2024.1404598

**Published:** 2024-07-23

**Authors:** Lisa Schwab, Jennifer L. Reichel, Antonia M. Werner, Markus Schäfer, Sebastian Heller, Dennis Edelmann, Manfred E. Beutel, Stephan Letzel, Pavel Dietz, Perikles Simon, Kristin Kalo

**Affiliations:** ^1^Department of Sports Medicine, Rehabilitation and Disease Prevention, Institute of Sport Science, Johannes Gutenberg University Mainz, Mainz, Germany; ^2^Institute of Occupational, Social and Environmental Medicine, University Medical Center of the University of Mainz, Mainz, Germany; ^3^Department of Psychosomatic Medicine and Psychotherapy, University Medical Center of the University of Mainz, Mainz, Germany; ^4^Department of Communication, Johannes Gutenberg University Mainz, Mainz, Germany; ^5^Institute for Applied Training Science, Strength, Power and Technical Sports, Leipzig, Germany

**Keywords:** laziness, sluggishness, sloth, health behavior, student health, health promotion

## Abstract

**Background:**

The transition from school to university is often accompanied by a change in students’ lifestyles. So far little is known whether convenience behavior is an essential factor affecting students’ health and social interaction. In a heterogeneous population regard to sociodemographic and anthropometric characteristics the Convenience Behavior Questionnaire (CBQ) showed a better relationship between convenience-related behavior with overweight and obesity than established questionnaires. Here we assessed convenience behavior in a large well characterized cohort of university students and its association with health-related (mainly sedentary behavior and physical activity), study-related and sociodemographic factors with the Convenience Behavior Questionnaire (CBQ).

**Methods:**

A total of 4,351 students participated in an online survey, of which 3,983 (23.6 ± 5.3 years old, 71.3% females) answered the questions concerning convenience behavior. A low value in the CBQ indicates more convenience behavior [Convenience Behavior Index (CBI) range: 3–15]. Differences with regard to sociodemographic (age, gender, body mass index), study-related (semester, degree, field of study) and health-related (physical activity, sedentary behavior) variables were examined with Mann–Whitney-U test or Kruskal–Wallis test and post-hoc tests (Bonferroni).

**Results:**

The CBI of men and women differed significantly (*z* = −6.847, *p* < 0.001, *r* = 0.11). First-year students and students beyond their first year showed significant differences (*z* = −2.355, *p* ≤ 0.05, *r* = 0.04). Differences were also found in the field of study (Chi2 (6) = 147.830, *p* < 0.001) and the targeted degree (Chi2 (7) = 79.985, *p* < 0.001). Furthermore, differences were found in the body mass index (Chi2 (5) = 70.678, *p* < 0.001), physical activity (Chi2 (2) = 279.040, *p* < 0.001) and sedentary behavior (*z* = −4.660, *p* < 0.001, *r* = 0.07).

**Conclusion:**

The results showed risk groups of convenience behavior among students [men, first-year, students enrolled in “Science, Technology, Engineering and Mathematics (STEM),” bachelor]. Our results confirm for the first time in a very homogeneous population a gender difference and an association between CBI and health-related factors. Further studies are needed to analyze the health behavior of students in more detail, especially their convenience behavior.

## Introduction

A person’s health and well-being depend on several factors such as genetic, environmental, or lifestyle factors. Lifestyle changes play a particularly important role in health promotion, as they are addressing modifiable risk factors (1). The most influential modifiable risk factors of an unhealthy lifestyle are physical inactivity, unbalanced diet, smoking, or excessive alcohol consumption (2, 3). These risk factors are associated with many chronic diseases such as obesity (4), cardiovascular diseases (5), or mental health issues (6, 7). Chronic diseases are considered the most important and most frequent causes of death and massively limit the quality of life of individuals (8, 9).

The term health behavior refers to the individual decisions, actions and habits that have a direct impact on a person’s health (10). These behaviors can be both positive and negative and encompass a variety of aspects that influence health. An unhealthy lifestyle, such as a lack of physical activity, high screen time, insufficient sleep or smoking, can occur especially during significant life changes. The transition from school to university represents such a life-changing event for young adults (11). Many university students move away from home and have to live on their own for the very first time. This new phase of life is often accompanied by lifestyle changes that have been shown to negatively affect sedentary behavior (SB) and physical activity (PA) (12, 13). SB is any waking behavior characterized by an energy expenditure of 1.5 metabolic equivalent of task (MET) or less while sitting, lying or standing (14). PA is any form of exercise that uses up energy (14). This includes structured physical activities such as sport and fitness exercises, but also everyday activities such as walking, climbing stairs or gardening. The daily university life can be demanding and is characterized by sedentariness, which occurs during lectures, while studying and researching (15, 16). Besides, university students often prefer watching TV or sitting at the computer instead of engaging in physical activity in their free time (16–18). In the study conducted by Carballo-Fazanes et al. (19), university students mentioned lack of time or laziness as the main reasons for not engaging in physical activity. Students’ social laziness is their unwillingness or inability to engage in healthy and productive social interactions in their home environment. For example, students prefer to engage in digital media rather than everyday social activities (20, 21).

Several studies have shown that a lower physical activity and a higher level of sedentary behavior are widespread among students (11, 15, 22, 23). In addition, the transition to higher education carries an increased risk of weight gain and negative changes in health behavior (24). A meta-analysis confirms a reciprocal relationship between depression and obesity (25). People who are overweight have a higher risk of developing depression. Furthermore, the meta-analysis shows that depression predicts the development of obesity (25). Factors such as changing lifestyles leading to eating disorders and academic worries can contribute to mental disorders that are common around university students all over the world (26, 27). Herewith, anxiety and mood disorders were the most prevalent classes of disorders (26). Depression is regarded as one of the most frequent health problems, affecting nearly a third of all students, which is higher than the reported prevalence in the general population (27).

In the context of behaviors that may contribute to an unhealthy lifestyle, the construct of convenience behaviors appears as an explanatory approach. Yet, there is no definition of convenience behavior. In our study, we define convenience behavior as the following patterns of behavior: Eating convenience or processed foods, avoiding conflicts, and preferring online contacts to face-to-face contacts, which also leads to them spending more time online. In addition, they are less physically active in everyday life and adopt an increasingly sedentary behavior. Dreher et al. (28) developed the Convenience Behavior Questionnaire (CBQ) to assess a Convenience Behavior Index (CBI) and established this index in many different collectives. The CBQ score is not directly related to a disease but refers to questions about convenience behavior in daily life. Convenience behavior includes behaviors that may contribute to an unhealthy lifestyle (29). The authors differentiated three dimensions (avoidance, social interaction, and domestic environmental behavior) of convenience behavior that can contribute to an unhealthy lifestyle in everyday life (28). The questions on avoidance behavior refer to avoiding arguments/conflicts, comparing oneself with peers in terms of convenience behavior and avoiding physical activity. The subcategory social interaction includes questions on social networks, willingness to meet in person, use of new media and intrinsic motivation to have new experiences. Domestic environmental behavior covers a wide range from eating habits and food safety/quality, social engagement to behaviors during vacation.

Regarding its association with health related variables the CBI was more strongly associated with overweight than physical activity assessed by a validated questionnaire (28). So far, there has been no research regarding the convenience behavior and its association with other health related factors than overweight. Furthermore, the CBQ was completed by a heterogeneous population (kindergarten teachers, primary school teachers, bus drivers, members of church choirs, parents of school children and parents of kindergarten children). University students were not included in the study by Dreher et al. (28). Students are a younger target group. Due to the conditions of study, it is a sensitive time which can contribute to an unhealthy lifestyle and many students struggle with health problems (30). Various studies have already established that age and gender can have an influence on health factors such as the development of obesity or mental health problems (31–33). Lifestyle, such as physical activity and sitting habits, can also have an influence on the development of diseases (34). Core symptoms of depression are a lack of energy, sleep disturbance and social withdrawal, which are likely to contribute to convenience behavior (35). The questions in the CBQ also deal with situations that can promote convenience behaviors and are common in students’ everyday lives.

For this reason, we aim to identify the level of convenience behavior of university students and assess its differences in sociodemographic (age, gender, body mass index), study-related (semester, degree, field of study) and health-related (PA, SB) factors. The results of this survey will help to identify risk groups of convenience behavior in university students. In the long term, the results might help to create more targeted interventions for health-promoting behaviors in students with regard to a convenience behavior. This would allow an intervention integrated into everyday life and adapted to behavioral patterns.

## Materials and methods

### Data collection and study design

In the summer semester of 2019, a cross-sectional online survey was conducted among all students at the Johannes-Gutenberg University of Mainz as part of an ongoing interdisciplinary project on student health promotion (“Healthy Campus Mainz”). All enrolled students (nearly 32,000) received an e-mail with an invitation to the online survey (36). A total number of 4,351 students participated in the survey, demonstrating a response rate of 13.9% of the university’s total student population at that time.

The Ethical Committee of the Medical Association of Rhineland-Palatinate approved the submitted study protocol (Number 2019-14336). Participation in the study was voluntary and informed consent was obtained at the beginning of the online survey.

### Measures

The questionnaire contained a wide range of health-related topics. Validated instruments were mainly used. An overview of all topics and items is given by Reichel et al. (36). The Convenience Behavior Questionnaire (CBQ) was used to record the Convenience Behavior Index (CBI) (28). The CBI can be divided into three subcategories: Avoidance Behavior Index (ABI), Social Interaction Behavior Index (SIBI), and Domestic Environment Index (DEI). The range of the CBI is from 3 to 15 while the subcategories range from 1 to 5. A low value in the CBI indicates convenience behaviors. Dreher et al. indicated the internal consistency of the CBI using Cronbach’s alpha as follows ABI (*α* = 0.801), SIBI (*α* = 0.891), and DEI (*α* = 0.854) (28). The CBQ is a newly developed questionnaire that depicts a new construct and, to our knowledge, has not yet been validated.

To determine differences in the CBI various sociodemographic (age, gender, body mass index), study-related (semester, degree, field of study) and health-related (PA, SB) variables were collected. The study-related variables are necessary to describe the sample and to identify risk groups. The body mass index (BMI) (kg/m^2^) was determined based on self-reported weight and height. The BMI was categorized according to the BMI classes defined by the WHO (37).

Participants who indicated that they were in their first or second university semester were classified as first-year students. Study subject and the degree were recorded and assigned to a corresponding field of study (“Science, Technology, Engineering and Mathematics”; “Social science, media or sport”; “Language, humanities or cultural sciences”; “Medicine”; “Law or economics”; “Aspiring teachers” or “Others”). For the target degree the following classification was made: “bachelor,” “master,” “dual degree,” “magister,” “state examination,” “diploma,” “Ph.D.” or “others.”

The German short form of the International Physical Activity Questionnaire – Short Form (IPAQ-SF) was used to query both physical activity and sitting time (38). The time spent sitting on a weekday was assessed as an indicator for sedentary behavior. According to the IPAQ-SF guidelines for data processing and analysis, questionnaires were considered invalid if any variable was missing or if the total amount of walking, moderate and vigorous activity exceeded 960 min (16 h) (39). The same procedure was followed for sitting time. Physical activity and the sitting time were recorded as minutes per day and days per week. Physical activity was divided into three groups: “insufficient physically active,” “moderate physically active” and “additional health benefits” (highly physically active). The classification was made according to the classification of the World Health Organization (WHO) (40). People who were moderately physically active for less than 150 min per week or vigorous physically active for less than 75 min per week were classified as insufficient physically active. Those who achieved the minimum requirement of 150 min of moderate or 75 min of vigorous physical activity were classified as moderate physically active, while those who achieved more than 300 min of moderate or 150 min of vigorous activity were classified as highly physically active. To create a binomial variable for sedentary behavior, a cut-off value of 8 h was set according to the literature (41). The IPAQ is a reliable and valid tool (38), and it is suitable as well as recommended to assess the physical activity level among university students (42).

### Data processing and statistical analyses

Descriptive statistics are presented as mean with standard deviation for continuous variables and as numbers and percentage from the total sample for non-continuous variables.

Data distribution was evaluated using Kolmogorov–Smirnov statistics with Lilliefors correction. Variance homogeneity was determined using the Levene test. Finally, non-parametric test procedures were chosen. Mann–Whitney-U-Test and Kruskal–Wallis-ANOVA with post-hoc test (Bonferroni) were used to evaluate differences in the convenience-related items regarding to sociodemographic, study-related and health-related factors.

Statistical analyses were performed using IBM SPSS Version 27 (IBM, Chicago, IL, USA). The significance level for all statistical analyses was set at *p* < 0.05.

## Results

A total of *N* = 3,983 participants answered the questions on convenience behavior and were included. All characteristics of the sample are summarized in Table 1. Due to the small group size, participants who indicated themselves as diverse (*n* = 32) were excluded from the main analyses. Mean age of the sample was 23.6 years (SD = 5.3 years), and 71.3% (*n* = 2,838) of the participants were female. In comparison to the gender distribution of the University of Mainz as a whole, in our study population women were overrepresented by 12.3% (43). The mean age was 23.6 years, which is comparable to the entire student body of the University of Mainz, where the mean age is 24.7 years. The majority of the sample are bachelor students with 52.3%, followed by students pursuing a state examination or a master’s degree.

**Table 1:** Characteristics of the study sample.

Variables	Value	Range
Sample size, *N*	3,983	
**Demographics**		
Age, *M* (SD)	23.6 (5.3)	16–73
Female, *n* (%)	2,838 (71.3)	
Male, *n* (%)	1,113 (27.9)	
Diverse, *n* (%)	32 (0.8)	
BMI (kg/m^2^), *M* (SD)	23.04 (4.2)	13.4–65.5
**Study characteristics**		
First year, *n* (%)	649 (16.3)	
University semester, *M* (SD)	7.2 (4.8)	1–45
**Degree**		
Bachelor, *n* (%)	2,082 (52.3)	
Master, *n* (%)	848 (21.3)	
Dual degree, *n* (%)	2 (0.1)	
Magister, *n* (%)	25 (0.6)	
State examination, *n* (%)	876 (22.0)	
Diploma, *n* (%)	3 (0.1)	
Ph.D., *n* (%)	138 (3.5)	
Others, *n* (%)	9 (0.2)	
**Field of study**		
STEM, *n* (%)	717 (18.0)	
Social science, media or sport, *n* (%)	717 (18.0)	
Language, humanities or cultural sciences, *n* (%)	803 (20.2)	
Medicine, *n* (%)	529 (13.3)	
Law or economics, *n* (%)	514 (12.9)	
Aspiring teachers, *n* (%)	613 (15.4)	
Others, *n* (%)	81 (2.0)	

STEM, Science, Technology, Engineering, and Mathematics.

BMI, Body Mass Index.

### Evaluation of convenience behavior and differences among students

In total, the analysis was carried out with 3,951 students. The mean values of the convenience behavior items are shown in Table 2. The mean score of the CBI was 10.06 (SD = 1.28). All sociodemographic (gender, age, body mass index), study-related (first-year, degree, field of study) and health-related (physical activity, sedentary behavior) characteristics of the participants are presented in Table 2. These are described in more detail in the following sub-chapters.

**Table 2:** Differences in sociodemographic, study-related and health-related factors in convenience behavior and the subcategories (ABI, SIBI, and DEI).

Variables	*n*	CBI		ABI		SIBI		DEI	
		*M* (SD)		*M* (SD)		*M* (SD)		*M* (SD)	
All	**3,951**	10.06 (1.28)		3.64 (0.65)		3.29 (0.49)		3.13 (0.61)	
**Sociodemographic factors**									
Gender”									
(a) Female	2,838	10.15 (1.26)	*z* = −6.847**; *r* = 0.11	3.66 (0.65)	*z* = −3.295**; *r* = 0.05	3.30 (0.48)	*z* = −2.654**; *r* = 0.04	3.19 (0.60)	*z* = −8.887**; *r* = 0.14
(b) Male	1,113	9.82 (1.28)		3.59 (0.65)		3.25 (0.50)		2.97 (0.61)	
Age”									
(a) Younger or equal 23 years	2,229	9.99 (1.27)	*z* = −4.060**; *r* = 0.06	3.59 (0.65)	*z* = −5.356**; *r* = 0.08	3.30 (0.50)	*z* = −1.895; *r* = 0.03	3.10 (0.61)	*z* = −4.156**; *r* = 0.07
(b) Older than 23 years	1,718	10.14 (1.28)		3.70 (0.64)		3.27 (0.48)		3.18 (0.60)	
BMI^#^									
(a) Underweight	233	9.95 (1.31)	*X*^2^ = 70.678**; c-b; d-b	3.68 (0.66)	*X*^2^ = 67.638**; e-b; e-a; d-b; d-a; c-a	3.19 (0.46)	*X*^2^ = 30.491**; a-b; c-d	3.08 (0.63)	*X*^2^ = 34.756**; c-b
(b) Normal weight	2,785	10.16 (1.24)		3.68 (0.62)		3.31 (0.49)		3.17 (0.60)	
(c) Pre-obesity	675	9.80 (1.32)		3.52 (0.67)		3.25 (0.49)		3.02 (0.61)	
(d) Obesity class I	153	9.63 (1.36)		3.42 (0.68)		3.19 (0.48)		3.02 (0.65)	
(e) Obesity class II	43	9.70 (1.28)		3.30 (0.75)		3.30 (0.56)		3.10 (6.11)	
(f) Obesity class III	30	9.51 (1.50)		3.31 (0.83)		3.03 (0.54)		3.17 (0.62)	
**Study-related factors**									
First year”									
(a) Yes	645	9.95 (1.24)	*z* = −2.355*; *r* = 0.04	3.58 (0.66)	*z* = −2.362*; *r* = 0.04	3.31 (0.49)	*z* = −1.074; *r* = 0.02	3.05 (0.61)	*z* = −3.586**; *r* = 0.06
(b) No	3,200	10.08 (1.28)		3.65 (0.65)		3.28 (0.49)		3.15 (0.61)	
Degree^***#***^									
(a) Bachelor	2,064	9.89 (1.27)	*X*^2^ = 79.985**; a-b; a-e; a-g	3.55 (0.66)	*X*^2^ = 93.804**; a-b; a-e; a-g	3.26 (0.49)	*X*^2^ = 21.374**; a-h	3.08 (0.61)	*X*^2^ = 35.688**; a-b; a-e
(b) Master	835	10.18 (1.22)		3.70 (0.61)		3.29 (0.48)		3.20 (0.59)	
(c) Dual Degree	2	10.61 (0.25)		3.88 (0.53)		3.40 (0.28)		3.33 (0.00)	
(d) Magister	25	9.85 (1.46)		3.47 (0.73)		3.31 (0.52)		3.07 (0.61)	
(e) State examination	875	10.29 (1.28)		3.77 (0.63)		3.34 (0.50)		3.18 (0.62)	
(f) Diploma	3	9.86 (1.45)		3.50 (0.90)		2.80 (0.40)		3.56 (0.19)	
(g) Ph.D.	138	10.29 (1.30)		3.80 (0.62)		3.28 (0.44)		3.20 (0.61)	
(h) Others	9	10.75 (0.99)		4.08 (0.43)		3.36 (0.48)		3.31 (0.61)	
Field of study^***#***^									
(a) STEM	714	9.76 (1.28)	*X*^2^ = 147.830**; a-b; a-d; a-e; a-f; c-b; c-d; c-e; c-f; e-d; b-d; f-d	3.55 (0.65)	*X*^2^ = 106.447** c-b; c-d; c-e; c-f; a-b; a-d; a-e; f-d; b-d; e-d	3.22 (0.51)	*X*^2^ = 49.083**; a-d; a-e; c-d; e-d; b-d	2.99 (0.61)	*X*^2^ = 96.392**; a-b; a-d; a-e; a-f; g-d; c-b; c-d; c-e; e-d
(b) Social sciences, media or sport	713	10.15 (1.20)		3.67 (0.60)		3.29 (0.46)		3.20 (0.61)	
(c) Language, humanities, or cultural sciences	783	9.81 (1.35)		3.51 (0.68)		3.24 (0.51)		3.06 (0.61)	
(d) Medicine	529	10.52 (1.22)		3.85 (0.61)		3.39 (0.47)		3.70 (0.61)	
(e) Law or economics	512	10.08 (1.22)		3.68 (0.63)		3.29 (0.49)		3.11 (0.60)	
(f) Aspiring teachers	611	10.19 (1.21)		3.65 (0.64)		3.32 (0.48)		3.22 (0.57)	
(g) Others	80	9.91 (1.30)		3.62 (0.72)		3.25 (0.48)		3.03 (0.62)	
**Health-related factors**									
Sedentary Behavior”									
(a) <8 h	2,020	10.16 (1.22)	*z* = −4.660**; *r* = 0.07	3.68 (0.62)	*z* = −4.201**; *r* = 0.07	3.31 (0.47)	*z* = −3.401**; *r* = 0.05	3.16 (0.61)	*z* = −2.506*; *r* = 0.04
(b) >=8 h	1,823	9.95 (1.32)		3.60 (0.67)		3.26 (0.51)		3.10 (0.61)	
Physical activity^***#***^									
(a) Insufficient physically active	863	9.43 (1.30)	*X*^2^ = 279.040**; a-b; a-c; b-c	3.38 (0.68)	*X*^2^ = 205.155** a-b; a-c; b-c	3.14 (0.50)	*X*^2^ = 105.055**; a-b; a-c; b-c	2.91 (0.60)	*X*^2^ = 146.880**; a-b; a-c; b-c
(b) Moderate physically active	688	10.02 (1.18)		3.60 (0.63)		3.28 (0.47)		3.14 (0.58)	
(c) Additional health benefits	2,344	10.30 (1.21)		3.75 (0.61)		3.34 (0.48)		3.21 (0.60)	

”Mann Whitney U-Test.

^#^Kruskal-Wallis with post-hoc tests Dunn–Bonferroni.

Alphabetic characters represent significant differences (*p* < 0.05) in the CBI and the subcategories ABI, SIBI, and DEI between respective categories of that variables.

M, Mean; SD, Standard deviation; CBI, Convenience Behavior Index; ABI, Avoidance Behavior Index; SIBI, Social Interaction Behavior Index; DEI, Domestic Environment Index; BMI, Body Mass Index; STEM, Science, Technology, Engineering and Mathematics.

*≤0.05, **≤0.001.

Insufficient physically active: less than 150 min moderate or 75 min vigorous per week.

Moderate physically active: more than 150 min moderate or 75 min vigorous per week.

Additional health benefits: more than 300 min moderate or 150 min vigorous per week.

#### Sociodemographic factors

Mean scores in females (10.15 ± 1.26) were higher than in males (9.82 ± 1.28). All indexes of the subcategories were significantly higher in females than in males. The effect size according to Cohen (44) is *r* = 0.11 and corresponds to a weak effect. For students younger than 23 years, the CBI was significantly lower than for older students. There were also differences with regard to BMI: normal-weight and pre-obesity individuals (*z* = 6.570; *p* < 0.001; *r* = 0.11) and between normal-weight and obesity individuals (*z* = 4.739; *p* < 0.001; *r* = 0.09). The mean and standard deviation of all variables as well as the results of Mann–Whitney-U-Test and Kruskal–Wallis with post-hoc comparisons (Dunn–Bonferroni) for the CBI and all three subcategories are depicted in Table 2.

#### Study-related factors

First-year students showed a lower score compared to higher semester students (Table 2). A bachelor’s degree was significantly different from a master (*z* = −5.262; *p* < 0.001, *r* = 0.10), state examination (*z* = −7.797, *p* < 0.001; *r* = 0.14) and a Ph.D. (*z* = −3.676, *p* < 0.001, *r* = 0.08) in terms of convenience behavior.

Students assigned to the field of study “Science, Technology, Engineering and Mathematics” (STEM) showed the most convenience behavior and differed significantly from the least convenience field of study “medicine” (*z* = −10.523; *p* < 0.001; *r* = 0.30). STEM was also significantly different from “social science, media or sport” (*z* = −5.716; *p* < 0.001; *r* = 0.15), “law and economics” (*z* = −3.987; *p* < 0.001; *r* = 0.11) and “aspiring teachers” (*z* = −5.796; *p* < 0.001; *r* = 0.16). The subject group “Language, humanities or cultural sciences” differed significantly from “social science, media or sport” (*z* = 4.955; *p* < 0.001; *r* = 0.13), “medicine” (*z* = −9.907; *p* < 0.001; *r* = 0.28), “law or economics” (*z* = −3.251; *p* = 0.02; *r* = 0.10), and aspiring teachers (*z* = −5.064; *p* < 0.001; *r* = 0.14.). “Law or Economics” and “medicine” were significantly different (*z* = 6.013; *p* < 0.001; *r* = 0.19). “Social science, media or sport” showed a significant difference to “medicine” (*z* = −5.246; *p* < 0.001; *r* = 0.15). “Aspiring teachers” also varied to “medicine” (*z* = 4.786; *p* < 0.001; *r* = 0.14).

#### Health-related factors

Students who used to sit more than 8 h per day showed a more convenience behavior (*z* = −4.660; *p* < 0.001; *r* = 0.07; Table 2) than students with a lower sitting time. Differences were also found with regard to physical activity. Physical inactive students had the lowest CBI and differed significantly from moderate physical active students (*z* = −3.827; *p* < 0.001; *r* = 0.14) and from students who were highly physically active (*z* = 7.630, *p* < 0.001; *r* = 0.16). Moderate physically active students also differed significantly from the highly physically active group (*z* = −4.455; *p* < 0.001; *r* = 0.10). The lowest values for physical activity and sedentary behavior were found in the DEI subcategory (Table 2).

## Discussion

The aim of the study was to examine student’s convenience-related behavior (avoidance, social interaction, domestic environmental behavior).

Male students had a significantly higher convenience behavior than female students. Younger students tended to more convenience behavior than their older peers. In addition, overweight students showed the most convenience behavior, while normal-weight students showed the least convenience behavior. With regard to significant differences in the study-related factors, it can be stated that first-year students exhibit more convenience behavior. Bachelor students and students from the field of study “Science, Technology, Engineering and Mathematics” (STEM) showed the highest convenience behavior. Students with low levels of physical activity and students who sit more than 8 h a day were more convenience than students with higher levels of physical activity or lower levels of sedentary behavior.

### Evaluation of convenience behavior and differences among students

The convenience behavior of university students is about the same level as that of the heterogeneous sample in the study by Dreher et al. (28). The average CBI was 10.6, which can be considered a medium to high level, and could be an indication of an unhealthy lifestyle among students. Regarding gender-specific aspects, male students showed more convenience behavior than females. These findings are consistent with the results of Dreher et al. (28), showing these gender differences in different population groups (28). Moreover, men use in general fewer behavioral strategies than women (45). In particular, social support is more often used by women than by men (46). This could therefore also be an explanation when looking at the subcategory SIBI (social interaction behavior index). Focusing on the field of study, “STEM” showed the most convenience behavior in this research. This could be due to the fact that men are overrepresented in this field of study. Another possible explanation mentioned in the literature is laziness or procrastination as behaviors that are common among students of these field of study (47).

In terms of study-related risk groups, bachelor students and first year students showed the most convenience behavior. A possible explanation could be that bachelor’s and first-year students are usually younger, and do not yet know how to manage the transition from school to university, and may therefore use avoidance strategies (48). In addition, it could be that there are fewer social contacts due to a change of residence. Especially students who live far away from their parents’ house tend to have unhealthy eating habits (49). These results could also explain why a higher BMI is associated with more convenience behavior.

With regard to the health-related variables, students who tend to sit longer per day or are less physically active show more convenience behavior. The students in the study by Carballo-Fazanes et al. (19) also reported laziness as one of the main reasons for physical inactivity. Edelmann et al. (50) pointed out that natural science students tend to sit longer during the day than other study groups. This group of students also had a more convenience behavior. Moreover, Huang (51) noted that students’ health awareness of eating habits and fresh ingredients is low, which could explain higher convenience behavior levels in the DEI subcategory. It can be summarized that students with a healthy lifestyle (measured by activity level and sitting time) show less convenience behavior.

### Practical relevance

Dreher et al. (28) showed that a low score in the CBI is associated with a higher BMI. Higher body weight in turn is related to chronic diseases (52, 53). In addition, students with a higher CBI included in our study appear to be more sedentary and less physically active. Therefore, the CBQ could be used as a measurement tool to identify specific risk groups with unhealthy behaviors, that lead to chronic diseases. Using the CBQ, we were able to identify such risk groups in the student population (men, field of study STEM, first year students) that are in line with previous studies investigating risk factors for an unhealthy lifestyle in students (50). Based on our results, target group-specific health interventions can be created. For example, the specific characteristics of the fields of study should be included. Starting from target group-specific results, the student health management could initiate first steps to improve the health behavior of students. This approach could lead to the development of suitable preventive measures. Studies have already provided initial evidence that interventions such as exercise programs or a physical breaks during the lecture (54), knowledge about healthy eating habits (55) and motivation programs (56) offer added health value. By creating suitable spaces and health-promoting time structures, the aim is to encourage health behavior (57).

As we have studied behaviors that lead to chronic disease rather than the symptoms of a disease itself, health interventions can be more effectively incorporated into everyday life by directly influencing unhealthy behaviors.

### Limitations and future research

Since participation in the questionnaire was voluntary, a certain selection bias in the sample cannot be excluded. Thus, it is possible that mainly people with high health behavior or specific behavior patterns participated in the study. Women were overrepresented in the study. These gender-specific differences in terms of response rates are a common phenomenon and are consistent with the research findings among students (58, 59). In addition, the BMI is based on self-reporting. The risk of self-reported data is often biased by social desirability. Height is often overestimated, whereas weight is underestimated (60). Furthermore, the questionnaire only covers the convenience behavior of university students at one university in Germany. To generalize the findings, the group differences in the CBI as well as the association with other health problems have to be measured at more universities in Germany or other countries.

So far, there is limited evidence on the convenience behavior of university students. It was the first attempt to apply the CBQ to the student sample. Currently, convenience behavior is based on an index without meaningful reference values. The questionnaire has not yet been validated. However, as the CBQ includes several constructs, the next steps should be to consider how a validation of the questionnaire would be possible. Since the questionnaire captures the behaviors holistically and is not limited to a specific disease, it could offer great potential for identifying risky health behaviors. Future studies should also validate the CBQ in populations with other established questionnaires on unhealthy behaviors. In this context, a classification to interpret convenience behavior should be conducted. With classification or cut-off values, convenience behaviors could be predicted in a more specific way.

As there are no other comparative values so far, an evaluation of convenience behavior can only be made within the framework of the present study. Furthermore, Dreher et al. (28) tested the CBQ on participants with different socio-economic backgrounds and social structures (e.g., kindergarten teachers, teachers, bus drivers, choir members, parents of school children, primary school teachers, parents of kindergarten children). Due to the small effect sizes, further investigations are necessary to verify the CBQ as a tool to identify unhealthy risk behaviors and to confirm the risk groups in university students, we have identified. Further studies could evaluate the association between convenience behavior and procrastination, learning difficulties or academic performance in university students. Procrastination among university students, for example, is a common problem and is due to poor time management, lack of planning for school activities, stress, but also laziness (61).

## Conclusion

To the best of our knowledge, this is the first study investigating convenience behavior among students. We revealed, that the CBI differs regarding sociodemographic, study-related and health-related factors. Male, first year and bachelor students as well as students in the field of study “Science, Technology, Engineering and Mathematics” could be identified as particular risk groups for convenience behavior. Students with a healthy lifestyle (measured by activity level and sitting time) show less convenience behavior. Based on these findings, the specific subgroups should be taken into account to develop specific health-promoting interventions in the university setting. In addition, CBQ can be used to design and implement specific strategies to change certain behaviors. Follow-up studies could validate the Convenience Behavior Questionnaire in the university setting.

## Data availability statement

The raw data supporting the conclusions of this article will be made available by the authors, without undue reservation.

## Ethics statement

The studies involving humans were approved by the Medical Association of Rhineland-Palatinate. The studies were conducted in accordance with the local legislation and institutional requirements. The participants provided their written informed consent to participate in this study.

## Author contributions

LS: Software, Methodology, Investigation, Formal analysis, Data curation, Conceptualization, Writing – review & editing, Writing – original draft. JR: Writing – review & editing, Data curation. AW: Writing – review & editing, Data curation. MS: Writing – review & editing, Data curation. SH: Writing – review & editing, Data curation. DE: Writing – review & editing, Data curation. MB: Writing – review & editing, Data curation. SL: Writing – review & editing, Resources, Project administration, Funding acquisition, Data curation. PD: Writing – review & editing, Resources, Project administration, Data curation, Conceptualization. PS: Writing – review & editing, Validation, Supervision, Investigation, Formal analysis, Data curation, Conceptualization. KK: Conceptualization, Writing – review & editing, Validation, Supervision, Software, Methodology, Investigation, Formal analysis, Data curation.
